# 2452. Prevalence of Antimicrobial ReSistanCE iN BloodStream Infections in HOspitalized (BSI) PatieNts in BRazil (ASCENSION-BR): Preliminary Results of a Multicenter Study

**DOI:** 10.1093/ofid/ofad500.2070

**Published:** 2023-11-27

**Authors:** Laura C Antochevis, Afonso Barth, Camila Wilhelm, Cláudia Carrilho, Beatriz Arns, Luiz Felipe Guimarães, Carlos E Starling, Andre Coelho, Elisa Mendes, Verônica Rocha, Amanda Martins, Evelyne Girão, João Telles, Letícia Sudbrack, Robson Leão, Alexandre Zavascki

**Affiliations:** Universidade Federal do Rio Grande do Sul, Porto Alegre, Rio Grande do Sul, Brazil; Hospital de Clínicas de Porto Alegre, Porto Alegre, Rio Grande do Sul, Brazil; Hospital de Clínicas de Porto Alegre, Porto Alegre, Rio Grande do Sul, Brazil; Universidade Estadual de Londrina, Londrina, Parana, Brazil; Hospital Moinhos de Vento, Porto Alegre, Rio Grande do Sul, Brazil; Hospital Universitário Clementino Fraga Filho, Rio de Janeiro, Rio de Janeiro, Brazil; Sociedade Mineira de Infectologia - SMI, Belo Horizonte, Minas Gerais, Brazil; Hospital Universitário de Brasília, Brasília, Distrito Federal, Brazil; Hospital PUC-Campinas, Campinas, Sao Paulo, Brazil; Instituto Couto Maia, Salvador, Bahia, Brazil; Hospital São Lucas, Porto Alegre, Rio Grande do Sul, Brazil; Hospital Universitário Walter Cantídio da Universidade Federal do Ceará, Fortaleza, Ceara, Brazil; Hospital Universitário Evangelico Mackenzie, Curitiba, Parana, Brazil; Hospital de Base do Distrito Federal, Brasília, Distrito Federal, Brazil; Hospital Universitário Pedro Ernesto/UERJ, Rio de Janeiro, Rio de Janeiro, Brazil; Hospital de Clínicas de Porto Alegre, Porto Alegre, Rio Grande do Sul, Brazil

## Abstract

**Background:**

Carbapenem-resistant *Enterobacterales* (CRE), *P. aeruginosa* (CRPA), *A. baumannii* (CRAB), methicillin-resistant *S. aureus* (MRSA) and vancomycin-resistant *E. faecium* (VRE) are major multidrug-resistant (MDR) bacteria causing healthcare-associated infections. There is no multicenter study addressing the prevalence of these MDR bacteria in Brazil. We report the preliminary results of the ongoing ASCENSION-BR study evaluating the prevalence of these microorganisms in BSI in hospitalized patients in this country.

**Methods:**

This prospective multicenter study has been conducted in 13 hospitals distributed in 4 of 5 Brazilian regions. From 15th August to 31st April (to be finished on 14th August 2023), each hospital recruited patients for a period of up to 6 months. Adult patients hospitalized for at least 48h, with positive blood cultures with *Enterobacterales, P. aeruginosa, A. baumannii*, *S. aureus* and *Enterococcus* spp. were eligible. More than one BSI of the same patients were included if caused by a distinct bacteria. MDR isolates were sent to a central laboratory where isolates were re-identified by MALDI-TOF-MS.

**Results:**

A total of 10 hospitals recruited patients for 6 months(m), 1 for 5m, and 2 for 1m. 1231 bacterias were isolated from 1091 BSI episodes (118 were polymicrobial), and 442 (35.9%) were MDR. Enterobacterales were the most common isolates in BSIs (659, 75.3%), followed by *S. aureus* (221, 18.0%), *A. baumannii* (113, 12.9%), *Enterococcus* spp. (135, 11.0%: 48 [35.6%] *E. faecium* and 84 [62.2%] *E. faecalis*) and *P. aeruginosa* (103, 8.4%). Among *Enterobacterales*, the most frequent species were *K. pneumoniae* (275, 41.7%), *E. coli* (126, 19.1) and *Enterobacter* spp. (69, 10.5%). A total of 224 (34.0% of Enterobacterales) BSIs were caused by CRE (155 [69.2%] *K. pneumoniae,* 27 [12.1%] *Serratia marcescen*s, 15 [6,7%] *Enterobacter* spp., and 27 [12.1%] other 11 species. A total of 100 (88.5% of *A. baumannii*) isolates recovered in BSIs were CRAB and 38 (36.9% of *P. aeruginosa*) were CRPA. Among Gram positives, 50 (22.6% of SA) were MRSA, and VRE represented 93.3% of *E. faecium* and 3.3% of *E. faecalis* isolates.

**Conclusion:**

High prevalence of MDR in BSI, notably in *A. baumannii*, were found among hospitalized patients in Brazil.

**Disclosures:**

**João Telles, MD, PhD**, MSD: Advisor/Consultant|MSD: Speaker|Pfizer: Advisor/Consultant|Pfizer: Speaker|União Química: Advisor/Consultant|União Química: Speaker **Alexandre Zavascki, MD, PhD**, Pfizer: Advisor/Consultant|Pfizer: Grant/Research Support

